# 1,2-Di­hydro­spiro­[carbazole-3(4*H*),2′-[1,3]dioxolane]

**DOI:** 10.1107/S1600536809005558

**Published:** 2009-02-21

**Authors:** Janni Vester Bjerrum, Trond Ulven, Andrew D. Bond

**Affiliations:** a University of Southern Denmark, Department of Physics and Chemistry, Campusvej 55, 5230 Odense M, Denmark

## Abstract

In the title compound, C_14_H_15_NO_2_, the hydrogenated six-membered ring of the carbazole unit adopts a half-chair conformation and the dioxolane ring points to one side of the carbazole plane. Neighbouring mol­ecules form edge-to-face inter­actions in which the NH group is directed towards an adjacent carbazole unit, with a shortest H⋯C contact of 2.72 Å. These inter­actions arrange the mol­ecules into one-dimensional herringbone-type motifs, which pack so that the methyl­ene groups of the dioxolane ring lie over the face of a neighbouring carbazole unit with a shortest H⋯C contact of 2.85 Å.

## Related literature

For background literature and synthesis details, see: Ulven & Kostenis (2006 ▶); Urrutia & Rodriguez (1999 ▶).
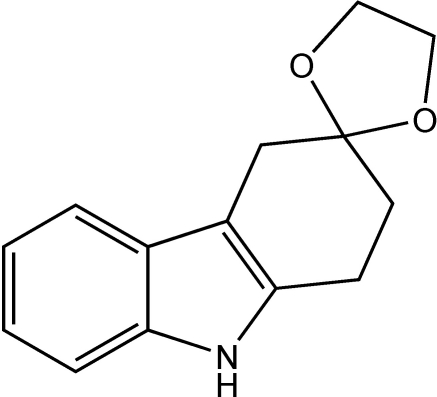


## Experimental

### 

#### Crystal data

C_14_H_15_NO_2_
*M* *_r_* = 229.27
Monoclinic, 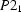

*a* = 9.3781 (6) Å
*b* = 6.1467 (4) Å
*c* = 10.5740 (7) Å
β = 115.232 (2)°
*V* = 551.38 (6) Å^3^
*Z* = 2
Mo *K*α radiation
μ = 0.09 mm^−1^
*T* = 180 K
0.50 × 0.50 × 0.40 mm

#### Data collection

Bruker–Nonius X8 APEXII CCD diffractometer
Absorption correction: multi-scan (*SADABS*; Sheldrick, 2003 ▶) *T*_min_ = 0.812, *T*_max_ = 0.964
7776 measured reflections
1485 independent reflections
1427 reflections with *I* > 2σ(*I*)
*R*_int_ = 0.017

#### Refinement

*R*[*F* ^2^ > 2σ(*F* ^2^)] = 0.029
*wR*(*F* ^2^) = 0.080
*S* = 1.05
1485 reflections
154 parameters
1 restraint
H-atom parameters constrained
Δρ_max_ = 0.33 e Å^−3^
Δρ_min_ = −0.16 e Å^−3^

### 

Data collection: *APEX2* (Bruker, 2004 ▶); cell refinement: *SAINT* (Bruker, 2003 ▶); data reduction: *SAINT*; program(s) used to solve structure: *SHELXS97* (Sheldrick, 2008 ▶); program(s) used to refine structure: *SHELXL97* (Sheldrick, 2008 ▶); molecular graphics: *SHELXTL* (Sheldrick, 2008 ▶); software used to prepare material for publication: *SHELXTL*.

## Supplementary Material

Crystal structure: contains datablocks global, I. DOI: 10.1107/S1600536809005558/ya2088sup1.cif

Structure factors: contains datablocks I. DOI: 10.1107/S1600536809005558/ya2088Isup2.hkl

Additional supplementary materials:  crystallographic information; 3D view; checkCIF report

## Figures and Tables

**Table 1:** Hydrogen-bond geometry (Å, °)

*D*—H⋯*A*	*D*—H	H⋯*A*	*D*⋯*A*	*D*—H⋯*A*
N1—H1*A*⋯C1^i^	0.88	2.72	3.527 (2)	154
C14—H14*A*⋯C12^ii^	0.99	2.85	3.518 (3)	126

Symmetry codes: (i) 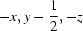; (ii) 
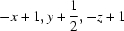
.

**Fig. 1.**
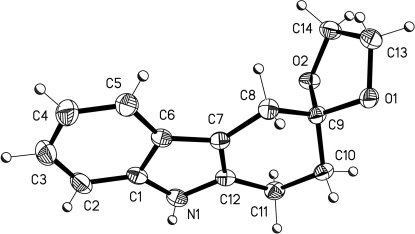
Molecular structure of the title compound with displacement ellipsoids shown at 50% probability for non-H atoms.

**Fig. 2.**
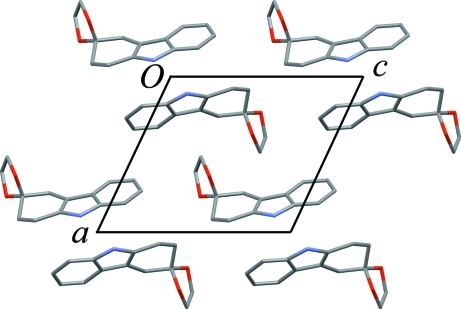
Projection along b showing interactions between carbazole units (e.g. about the origin), and between dioxolane rings and carbazole units (e.g. at the centre of the unit cell). H atoms are omitted.

## supplementary crystallographic information

### Comment

The title compound is useful as an intermediate in the synthesis of antagonists of the prostaglandin D_2_ receptor CRTH2 (DP_2_) (Ulven & Kostenis, 2006).

### Experimental

The compound was synthesized as described in Urrutia & Rodriguez (1999).

### Refinement

H atoms bound to C atoms were placed in idealized positions with C—H = 0.95 or 0.99 Å and refined as riding with *U*_iso_(H) = 1.2*U*_eq_(C). The methyl group was allowed to rotate about its local threefold axis. The H atom of the NH group was visible in a difference Fourier map but was placed geometrically and refined as riding for the final cycles of refinement with N—H = 0.88 Å and *U*_iso_(H) = 1.2*U*_eq_(N). In the absence of significant anomalous scattering, 1128 Friedel pairs were merged as equivalent data.

### Crystal data

**Table:**

C_14_H_15_NO_2_	*F*(000) = 244
*M**_r_* = 229.27	*D*_x_ = 1.381 Mg m^−^^3^
Monoclinic, *P*2_1_	Mo *K*α radiation, λ = 0.71073 Å
Hall symbol: P 2yb	Cell parameters from 5883 reflections
*a* = 9.3781 (6) Å	θ = 2.4–28.4°
*b* = 6.1467 (4) Å	µ = 0.09 mm^−^^1^
*c* = 10.5740 (7) Å	*T* = 180 K
β = 115.232 (2)°	Block, colourless
*V* = 551.38 (6) Å^3^	0.50 × 0.50 × 0.40 mm
*Z* = 2	

### Data collection

**Table:**

Bruker–Nonius X8 APEXII CCD diffractometer	1485 independent reflections
Radiation source: fine-focus sealed tube	1427 reflections with *I* > 2σ(*I*)
graphite	*R*_int_ = 0.017
Thin–slice ω and φ scans	θ_max_ = 28.4°, θ_min_ = 3.9°
Absorption correction: multi-scan (*SADABS*; Sheldrick, 2003)	*h* = −12→12
*T*_min_ = 0.812, *T*_max_ = 0.964	*k* = −8→8
7776 measured reflections	*l* = −11→14

### Refinement

**Table:**

Refinement on *F*^2^	Primary atom site location: structure-invariant direct methods
Least-squares matrix: full	Secondary atom site location: difference Fourier map
*R*[*F*^2^ > 2σ(*F*^2^)] = 0.029	Hydrogen site location: inferred from neighbouring sites
*wR*(*F*^2^) = 0.080	H-atom parameters constrained
*S* = 1.05	*w* = 1/[σ^2^(*F*_o_^2^) + (0.0581*P*)^2^ + 0.0517*P*] where *P* = (*F*_o_^2^ + 2*F*_c_^2^)/3
1485 reflections	(Δ/σ)_max_ < 0.001
154 parameters	Δρ_max_ = 0.33 e Å^−^^3^
1 restraint	Δρ_min_ = −0.16 e Å^−^^3^

### Special details

**Table:**

Geometry. All e.s.d.'s (except the e.s.d. in the dihedral angle between two l.s. planes) are estimated using the full covariance matrix. The cell e.s.d.'s are taken into account individually in the estimation of e.s.d.'s in distances, angles and torsion angles; correlations between e.s.d.'s in cell parameters are only used when they are defined by crystal symmetry. An approximate (isotropic) treatment of cell e.s.d.'s is used for estimating e.s.d.'s involving l.s. planes.
Refinement. Refinement of *F*^2^ against ALL reflections. The weighted *R*-factor *wR* and goodness of fit *S* are based on *F*^2^, conventional *R*-factors *R* are based on *F*, with *F* set to zero for negative *F*^2^. The threshold expression of *F*^2^ > σ(*F*^2^) is used only for calculating *R*-factors(gt) *etc*. and is not relevant to the choice of reflections for refinement. *R*-factors based on *F*^2^ are statistically about twice as large as those based on *F*, and *R*- factors based on ALL data will be even larger.

### Fractional atomic coordinates and isotropic or equivalent isotropic displacement parameters (Å^2^)

**Table:**

	*x*	*y*	*z*	*U*_iso_*/*U*_eq_	
O1	0.23032 (12)	0.56670 (18)	0.55448 (11)	0.0256 (2)	
O2	0.36610 (11)	0.26085 (18)	0.56040 (10)	0.0238 (2)	
N1	0.10724 (14)	−0.0286 (2)	0.12685 (12)	0.0259 (3)	
H1A	0.0579	−0.1545	0.1083	0.031*	
C1	0.17592 (15)	0.0682 (3)	0.04893 (14)	0.0240 (3)	
C2	0.18695 (18)	−0.0023 (3)	−0.07194 (15)	0.0321 (3)	
H2A	0.1415	−0.1362	−0.1153	0.038*	
C3	0.2665 (2)	0.1299 (4)	−0.12652 (16)	0.0373 (4)	
H3A	0.2752	0.0863	−0.2092	0.045*	
C4	0.3344 (2)	0.3267 (3)	−0.06233 (18)	0.0368 (4)	
H4A	0.3891	0.4134	−0.1017	0.044*	
C5	0.32330 (18)	0.3972 (3)	0.05741 (15)	0.0296 (3)	
H5A	0.3694	0.5312	0.1000	0.036*	
C6	0.24301 (15)	0.2675 (3)	0.11475 (13)	0.0223 (3)	
C7	0.21109 (15)	0.2855 (2)	0.23567 (13)	0.0206 (3)	
C8	0.25187 (17)	0.4636 (2)	0.34150 (14)	0.0229 (3)	
H8A	0.1820	0.5900	0.3001	0.027*	
H8B	0.3620	0.5107	0.3687	0.027*	
C9	0.23321 (15)	0.3857 (2)	0.47164 (14)	0.0202 (3)	
C10	0.08364 (15)	0.2549 (3)	0.43550 (14)	0.0235 (3)	
H10A	−0.0086	0.3484	0.3823	0.028*	
H10B	0.0775	0.2110	0.5231	0.028*	
C11	0.07498 (17)	0.0513 (2)	0.34942 (15)	0.0249 (3)	
H11A	0.1428	−0.0641	0.4112	0.030*	
H11B	−0.0347	−0.0034	0.3056	0.030*	
C12	0.12847 (15)	0.1046 (2)	0.23882 (13)	0.0217 (3)	
C13	0.39081 (17)	0.6225 (3)	0.63924 (16)	0.0275 (3)	
H13A	0.4055	0.6671	0.7340	0.033*	
H13B	0.4255	0.7426	0.5965	0.033*	
C14	0.48246 (17)	0.4138 (3)	0.64530 (17)	0.0303 (3)	
H14A	0.5615	0.4393	0.6079	0.036*	
H14B	0.5372	0.3606	0.7427	0.036*	

### Atomic displacement parameters (Å^2^)

**Table:**

	*U*^11^	*U*^22^	*U*^33^	*U*^12^	*U*^13^	*U*^23^
O1	0.0236 (5)	0.0230 (5)	0.0319 (5)	0.0007 (4)	0.0134 (4)	−0.0055 (4)
O2	0.0198 (4)	0.0193 (5)	0.0280 (5)	0.0008 (4)	0.0061 (4)	0.0010 (4)
N1	0.0248 (6)	0.0226 (6)	0.0263 (6)	−0.0051 (5)	0.0071 (5)	−0.0031 (5)
C1	0.0195 (6)	0.0260 (7)	0.0206 (6)	0.0007 (5)	0.0029 (5)	0.0009 (5)
C2	0.0297 (7)	0.0371 (9)	0.0223 (6)	0.0005 (7)	0.0042 (5)	−0.0049 (6)
C3	0.0367 (8)	0.0505 (11)	0.0229 (6)	0.0035 (8)	0.0110 (6)	0.0002 (7)
C4	0.0379 (8)	0.0461 (10)	0.0293 (7)	−0.0009 (8)	0.0171 (7)	0.0055 (7)
C5	0.0315 (7)	0.0307 (7)	0.0266 (6)	−0.0033 (7)	0.0123 (6)	0.0037 (6)
C6	0.0200 (5)	0.0221 (6)	0.0209 (6)	0.0013 (5)	0.0049 (5)	0.0021 (5)
C7	0.0188 (5)	0.0195 (6)	0.0217 (6)	0.0003 (5)	0.0069 (5)	0.0024 (5)
C8	0.0271 (6)	0.0168 (6)	0.0263 (6)	−0.0022 (5)	0.0130 (5)	0.0012 (5)
C9	0.0192 (5)	0.0169 (6)	0.0251 (6)	0.0009 (5)	0.0100 (5)	−0.0009 (5)
C10	0.0191 (6)	0.0245 (7)	0.0283 (6)	−0.0021 (5)	0.0113 (5)	0.0002 (6)
C11	0.0246 (6)	0.0212 (7)	0.0303 (7)	−0.0056 (5)	0.0128 (5)	−0.0005 (5)
C12	0.0186 (5)	0.0195 (6)	0.0239 (6)	−0.0004 (5)	0.0062 (5)	0.0009 (5)
C13	0.0284 (7)	0.0233 (7)	0.0283 (6)	−0.0032 (6)	0.0099 (6)	−0.0020 (5)
C14	0.0227 (6)	0.0304 (8)	0.0323 (7)	−0.0011 (6)	0.0065 (6)	−0.0065 (6)

### Geometric parameters (Å, °)

**Table:**

O1—C9	1.4234 (17)	C7—C12	1.3639 (19)
O1—C13	1.4270 (17)	C7—C8	1.4936 (19)
O2—C9	1.4233 (16)	C8—C9	1.5358 (18)
O2—C14	1.4303 (18)	C8—H8A	0.990
N1—C1	1.3785 (19)	C8—H8B	0.990
N1—C12	1.3822 (18)	C9—C10	1.5177 (18)
N1—H1A	0.880	C10—C11	1.529 (2)
C1—C2	1.395 (2)	C10—H10A	0.990
C1—C6	1.417 (2)	C10—H10B	0.990
C2—C3	1.384 (3)	C11—C12	1.4922 (18)
C2—H2A	0.950	C11—H11A	0.990
C3—C4	1.400 (3)	C11—H11B	0.990
C3—H3A	0.950	C13—C14	1.530 (2)
C4—C5	1.383 (2)	C13—H13A	0.990
C4—H4A	0.950	C13—H13B	0.990
C5—C6	1.400 (2)	C14—H14A	0.990
C5—H5A	0.950	C14—H14B	0.990
C6—C7	1.4364 (18)		
			
C9—O1—C13	106.42 (10)	O2—C9—C10	109.62 (11)
C9—O2—C14	106.18 (11)	O1—C9—C10	108.13 (11)
C1—N1—C12	108.84 (12)	O2—C9—C8	110.90 (10)
C1—N1—H1A	125.6	O1—C9—C8	110.32 (11)
C12—N1—H1A	125.6	C10—C9—C8	112.68 (11)
N1—C1—C2	130.52 (15)	C9—C10—C11	113.10 (11)
N1—C1—C6	107.60 (12)	C9—C10—H10A	109.0
C2—C1—C6	121.87 (14)	C11—C10—H10A	109.0
C3—C2—C1	117.59 (16)	C9—C10—H10B	109.0
C3—C2—H2A	121.2	C11—C10—H10B	109.0
C1—C2—H2A	121.2	H10A—C10—H10B	107.8
C2—C3—C4	121.30 (15)	C12—C11—C10	109.68 (12)
C2—C3—H3A	119.3	C12—C11—H11A	109.7
C4—C3—H3A	119.3	C10—C11—H11A	109.7
C5—C4—C3	121.20 (16)	C12—C11—H11B	109.7
C5—C4—H4A	119.4	C10—C11—H11B	109.7
C3—C4—H4A	119.4	H11A—C11—H11B	108.2
C4—C5—C6	118.84 (16)	C7—C12—N1	109.75 (12)
C4—C5—H5A	120.6	C7—C12—C11	125.61 (13)
C6—C5—H5A	120.6	N1—C12—C11	124.58 (13)
C5—C6—C1	119.19 (13)	O1—C13—C14	104.37 (12)
C5—C6—C7	134.15 (14)	O1—C13—H13A	110.9
C1—C6—C7	106.65 (12)	C14—C13—H13A	110.9
C12—C7—C6	107.16 (13)	O1—C13—H13B	110.9
C12—C7—C8	123.08 (12)	C14—C13—H13B	110.9
C6—C7—C8	129.75 (12)	H13A—C13—H13B	108.9
C7—C8—C9	110.64 (11)	O2—C14—C13	105.08 (11)
C7—C8—H8A	109.5	O2—C14—H14A	110.7
C9—C8—H8A	109.5	C13—C14—H14A	110.7
C7—C8—H8B	109.5	O2—C14—H14B	110.7
C9—C8—H8B	109.5	C13—C14—H14B	110.7
H8A—C8—H8B	108.1	H14A—C14—H14B	108.8
O2—C9—O1	104.87 (11)		
			
C12—N1—C1—C2	179.41 (15)	C13—O1—C9—O2	36.02 (13)
C12—N1—C1—C6	0.36 (15)	C13—O1—C9—C10	152.93 (12)
N1—C1—C2—C3	−178.93 (15)	C13—O1—C9—C8	−83.44 (13)
C6—C1—C2—C3	0.0 (2)	C7—C8—C9—O2	79.85 (14)
C1—C2—C3—C4	0.4 (3)	C7—C8—C9—O1	−164.41 (11)
C2—C3—C4—C5	−0.6 (3)	C7—C8—C9—C10	−43.46 (16)
C3—C4—C5—C6	0.3 (3)	O2—C9—C10—C11	−64.65 (14)
C4—C5—C6—C1	0.2 (2)	O1—C9—C10—C11	−178.44 (11)
C4—C5—C6—C7	178.70 (16)	C8—C9—C10—C11	59.37 (16)
N1—C1—C6—C5	178.86 (13)	C9—C10—C11—C12	−42.89 (16)
C2—C1—C6—C5	−0.3 (2)	C6—C7—C12—N1	0.49 (15)
N1—C1—C6—C7	−0.06 (15)	C8—C7—C12—N1	179.18 (12)
C2—C1—C6—C7	−179.21 (13)	C6—C7—C12—C11	177.82 (13)
C5—C6—C7—C12	−178.94 (16)	C8—C7—C12—C11	−3.5 (2)
C1—C6—C7—C12	−0.27 (14)	C1—N1—C12—C7	−0.54 (15)
C5—C6—C7—C8	2.5 (3)	C1—N1—C12—C11	−177.90 (13)
C1—C6—C7—C8	−178.84 (13)	C10—C11—C12—C7	16.12 (19)
C12—C7—C8—C9	16.67 (18)	C10—C11—C12—N1	−166.94 (12)
C6—C7—C8—C9	−164.96 (13)	C9—O1—C13—C14	−22.68 (15)
C14—O2—C9—O1	−34.81 (13)	C9—O2—C14—C13	20.17 (14)
C14—O2—C9—C10	−150.69 (12)	O1—C13—C14—O2	1.51 (16)
C14—O2—C9—C8	84.26 (13)		

### Hydrogen-bond geometry (Å, °)

**Table:**

*D*—H···*A*	*D*—H	H···*A*	*D*···*A*	*D*—H···*A*
N1—H1A···C1^i^	0.88	2.72	3.527 (2)	154
C14—H14A···C12^ii^	0.99	2.85	3.518 (3)	126

Symmetry codes: (i) −*x*, *y*−1/2, −*z*; (ii) −*x*+1, *y*+1/2, −*z*+1.
